# Genome analysis of cotton leafroll dwarf virus reveals variability in the silencing suppressor protein, genotypes and genomic recombinants in the USA

**DOI:** 10.1371/journal.pone.0252523

**Published:** 2021-07-07

**Authors:** Afsha Tabassum, Sudeep Bag, Nelson D. Suassuna, Kassie N. Conner, Peng Chee, Robert C. Kemerait, Phillip Roberts

**Affiliations:** 1 Department of Plant Pathology, University of Georgia, Tifton, Georgia, United States of America; 2 Embrapa Algodão, Santo Antônio de Goiás, GO, Brazil; 3 Alabama Cooperative Extension System, Auburn University, Auburn, Alabama, United States of America; 4 Institute of Plant Breeding, Genetics and Genomics, University of Georgia, Tifton, Georgia, United States of America; 5 Department of Entomology, University of Georgia, Tifton, Georgia, United States of America; USDA Agricultural Research Service, UNITED STATES

## Abstract

Cotton leafroll dwarf virus (CLRDV) is an emerging virus in cotton production in Georgia and several other Southeastern states in the USA. To better understand the genetic diversity of the virus population, the near complete genome sequences of six isolates from Georgia and one from Alabama were determined. The isolates sequenced were 5,866 nucleotides with seven open reading frames (ORFs). The isolates from Georgia were >94% identical with other isolates from the USA and South America. In the silencing suppressor protein (P0), at amino acid position 72, the isolates from Georgia and Alabama had a valine (V), similar to resistant-breaking ‘atypical’ genotypes in South America, while the Texas isolate had isoleucine (I), similar to the more aggressive ‘typical’ genotypes of CLRDV. At position 120, arginine (R) is unique to Georgia and China isolates, but absent in Alabama, Texas and South American isolates. Ten potential recombinant events were detected in the isolates sequenced. An increased understanding of CLRDV population structure and genetic diversity will help develop management strategies for CLRDV in the USA cotton belt.

## 1 Introduction

Cotton (*Gossypium hirsutum* L.) is one of the most economically important crops grown in the Southeastern USA and has a farm gate value of over $792 million in 2018 [1]. However, cotton production can be negatively affected by several pathogens worldwide. Cotton leafroll dwarf virus (CLRDV) (Genus: *Polerovirus*; Family: *Luteoviridae*) is known to cause the devastating cotton blue disease (CBD) in Africa, Asia, and South America. The disease was first described in 1949 in the Central African Republic [2, 3]. CBD was named for the dark green to bluish color, inward rolling, and leathery texture of leaves on the infected plants. In early-season infections, epinasty can be severe, with reddened petioles and veins, and pronounced stunting of plants [2]. In 1938 ‘Vein Mosaic’ disease was observed in Brazil exhibiting similar symptoms. A more severe occurrence of the same disease, Vein Mosaic var. "Ribeirão Bonito" [4] was reported to cause economic losses. Observation of similar symptoms and vector (*Aphis gossypii*) transmission studies strongly suggested that vein mosaic and CBD had the same etiology [3]. Based on the partial sequence of the viral genome from symptomatic plants, the virus was determined to be a member of the genus *Polerovirus* [3]. Subsequently, the full-length genome of CLRDV was sequenced and characterized [5]. In 2006, although a less aggressive resistant-breaking genotype of CLRDV was observed in Brazil on cotton varieties known to be resistant against CBD [6]. This new disease was referred to as ‘atypical’ cotton blue disease (ACBD) or atypical vein mosaic disease to differentiate from the ‘typical’ CBD [6].

CLRDV contains a monopartite, single-stranded, positive-sense RNA of approximately 5.7kb in length with VPg at 5’ end and no poly(A) tail or t-RNA structure at 3’ end. The virus genome consists of seven open reading frames (ORFs) with an intergenic region between ORFs 2 and ORFs 3a. The ORF0 encodes the P0 protein (28.9kDa), which acts as a silencing suppressor [7, 10]. ORF1 encodes P1 protein (70.1kDa), predicted to be expressed through leaky scanning. ORF1-2 encodes fused protein P1-P2 replication-related protein of 118.7kDa through ribosomal frameshift. ORFs 3–5 express through sub-genomic RNAs. P3 (22.4kDa) encodes the coat protein, P4 (19.4kDa) encodes the movement protein, and P5 is translated through an in-frame read through P3 stop codon. P3-P5 (77.2kDa) is essential for aphid transmission and virus accumulation in plants [5, 8, 9]. The silencing suppressor protein P0 (ORF0) is the most variable genomic region [10, 11] and has an F-box domain LPxx(L/I)x_10-13_P essential for viral silencing suppressor activity [12, 13]. The ‘typical’ genotype of CLRDV can be differentiated from the ‘atypical’ by a single amino acid substitution of isoleucine (I) to valine (V) at position 72 [8].

In addition to Africa and South America, CLRDV has also been reported in Timor-Leste [14], Thailand [15], India [16], and from Soybean aphid (*Aphis glycines*) in China [17]. Recently, there have been several reports on the detection of CLRDV in the USA: Arkansas [18], Alabama [19], Florida [20], Georgia [21], Kansas [22], Louisiana [23], Mississippi [24], North Carolina [25], Oklahoma [26], South Carolina [27] and Texas [28]. Since the first report of CLRDV, it has been detected in all the major cotton-growing counties in Georgia with 0–30% of disease incidence.

CLRDV is considered an emerging disease in cotton in the USA. Although the viral genome of individual isolates each from Alabama [29], Georgia [30], and Texas [31] have been sequenced, no information is available on the genetic diversity and population structure of the virus. In this study, the nearly complete genome of six isolates from Georgia and one isolate from Alabama were sequenced, representing diverse symptoms, plant growth stage, and geographical locations to understand the genome variability of CLRDV in the USA. The information generated will provide a further understanding of the population structure and genetic diversity of the virus. This can be used in developing integrated management approaches, resistance breeding to reduce the impact of the disease.

## 2 Materials and methods

### 2.1 Virus isolates

**Fig 1:**
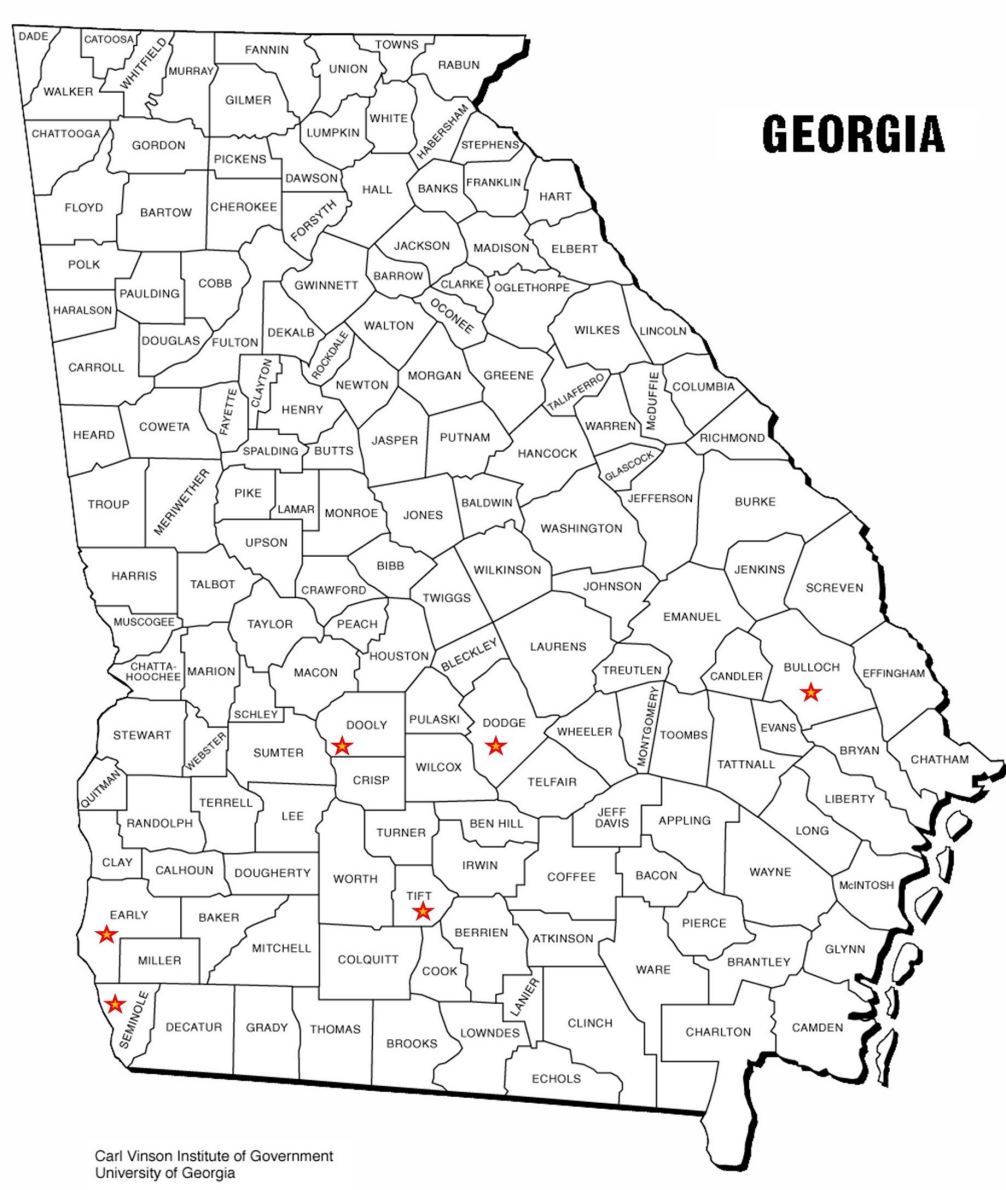
Map of Georgia, USA with the locations for sample collections shown in stars.

**Fig 2:**
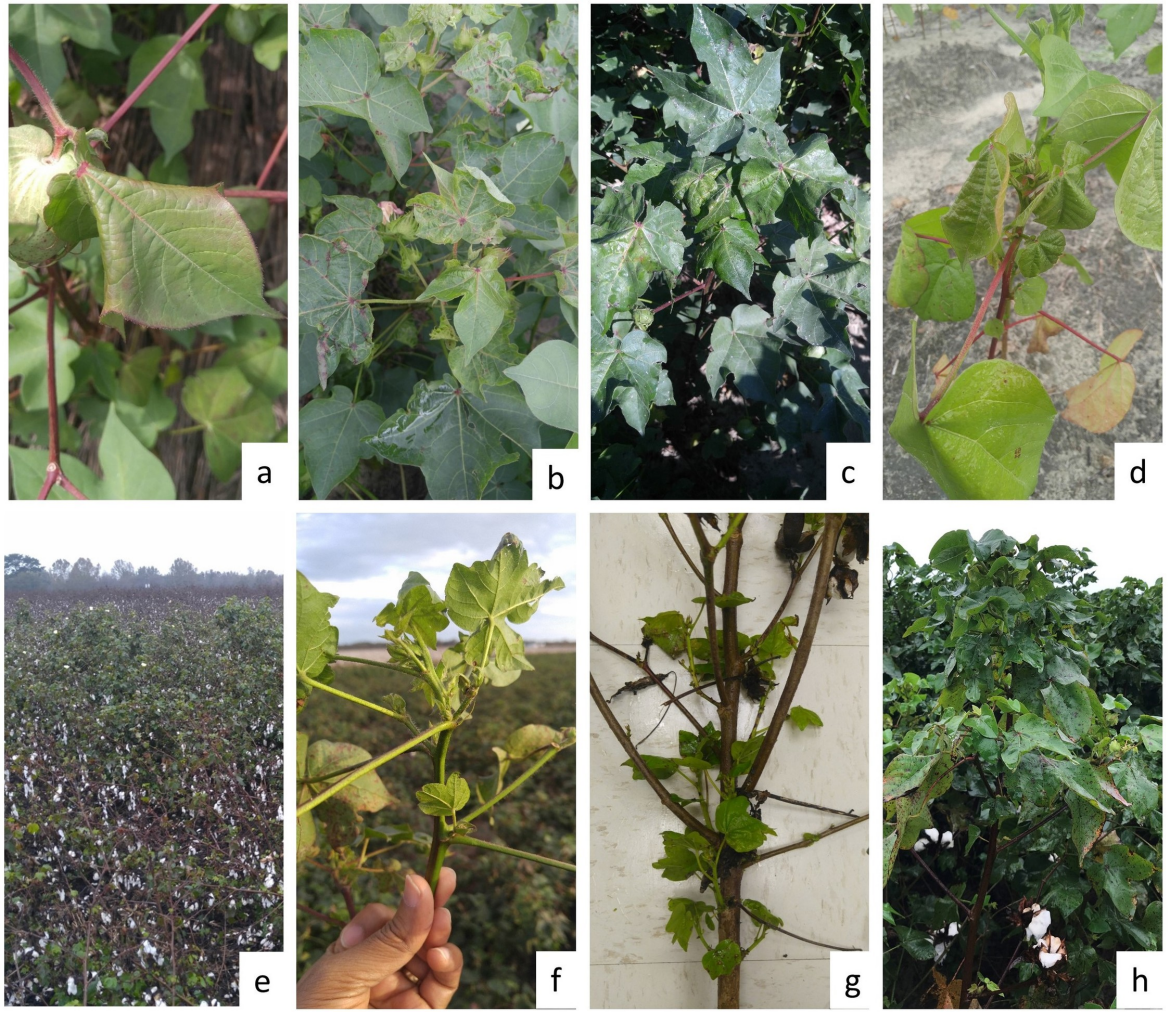
Isolates of cotton leafroll dwarf virus used in this study were collected from different counties of Alabama and Georgia, USA showing diverse symptoms including reddening and downward curling of leaf (a) and leaf distortion (b) from Tifton, Tift County, GA; leaf distortion and red petiole (c) from Seminole County, GA; reddening of leaf and petiole, leaf drooping (d) Brookfield, Tift County, GA; basal regrowth and extended growth (e) Bulloch County, GA; shortened internode (f) Dooly County, GA; basal regrowth (g) Dodge County, GA; leaf deformation, bunching top, leathery texture (h) Baldwin County, AL.

Cotton plants with distorted and deformed leaves, vein yellowing, leaf curl, reddening of leaves, petioles, stunted growth, and shortened internodes suggestive of CLRDV infection were collected from different cotton-growing counties of Georgia, USA (Fig 1). Symptomatic cotton plants exhibiting diverse symptoms were collected from multiple locations at two different crop growth stages (Table 1). Initially, the samples with red leaves, red petioles, and inverted ‘V’ shaped leaves were collected from Early, Seminole, and Tift counties in July-September 2018 (Fig 2A–2D). Later in November 2018, symptomatic tissue from plants with basal regrowth, shortened internodes, distorted growth, and leaf curling were collected from Bulloch, Dodge, and Dooly counties (Fig 2E–2G). A plant sample with downward foliar cupping, puckering, bunching, and leathery texture (Fig 2H) was collected from Fairhope, Baldwin County, Alabama. The samples collected from the symptomatic plants were stored at -80°C until further processing.

**Table 1:** List of cotton leafroll dwarf virus isolates collected and sequenced in this study.

Location (County, State)	GenBank Accession number	Symptoms	Date of collection
Bulloch, GA	MT800933	basal regrowth and extended growth	11/14/2018
Dodge, GA	MT814775	Basal regrowth	11/14/2018
Dooly, GA	MT814774	Shortened internodes	11/14/2018
Seminole, GA	MT633122	Leaf distortion and red petiole	09/19/2018
Brookfield, Tift, GA	MT814776	Red leaves and petioles, drooping	08/16/2018
Tifton, Tift, GA	MT800932	Leaf deformation, curling, red leaves and petiole	07/18/2018
Baldwin, AL	MT814777	Leaf deformation, bunching top, leathery texture	11/08/2018

### 2.2 Total RNA extraction, RT-PCR, cloning, and sequencing

Total RNA was extracted from 100 mg leaves, petioles, and stems tissues of symptomatic plants using the modified cetyltrimethylammonium bromide method [15, 32]. Complementary DNA (cDNA) was synthesized from 1μg of total RNA using Superscript III reverse transcription (Invitrogen, USA) and specific reverse primers targeting different open reading frames (ORFs) of the virus genome following the manufacturer’s recommended conditions. The cDNA (2μl) was used for polymerase chain reaction (PCR) with primers targeting different ORFs of the CLRDV genome [30]. PCR reactions were performed by Platinum Taq Green Hot Start DNA polymerase (Invitrogen, USA), using various primer combinations [30]. The PCR products of the expected size were gel purified, cloned into pGEM-T easy I cloning vector (Promega, USA), and sequenced using Sanger sequencing (GenScript, USA). Three clones from each amplicon were sequenced, and a consensus sequence was obtained. A nearly-complete nucleotide sequence was assembled and annotated using BioEdit software [33] and submitted to NCBI-GenBank (Table 1).

In 2018, 80 samples collected from commercial fields and UGA research farms in Georgia were tested for the presence of CLRDV using CLRDV3675F and Pol3982R [15] and SB11F/R primers [30] that amplify 310 nt and 775 nt, respectively, from ORF3 and ORF4. A total of six samples collected from different counties of Georgia and one sample from Baldwin County, Alabama, were used for near complete genome sequencing and characterization. The isolates were selected based on the symptoms including red leaves, red petioles, shortened internodes and leaf curling, location, and plant growth stages (Fig 2 and Table 1).

### 2.3 Genome analysis: Conserved motifs, pairwise identity, and phylogenetic analyses

The ClustalW alignment in BioEdit software [33] was used to identify the conserved motifs in predictive proteins. Pairwise comparisons between CLRDV isolates were performed with SDT v.2.1 [34]. Multiple Sequence Alignment (MSA) of all genome sequence were performed using MUSCLE [35] algorithm and built in MEGA X software [36]. The optimal nucleotide and amino acid substitution models were determined in MEGA X based on Bayesian information criterion (BIC) and Akaike information criterion (AIC). Kimura 2-parameter and Jones Taylor-Thornton were identified as the optimal evolutionary models for the nucleotide and amino acid sequences, respectively. Aligned sequence relatedness was evaluated using the Maximum Likelihood method (default parameters with 2000 bootstrap replicates) [36]. The cut-off value for the phylogenetic tree was 50%. CLRDV genomes reported across the globe were downloaded from the nucleotide sequence repository, GenBank, and were used in the construction of phylogenetic trees based on nucleotide and amino acid sequences.

### 2.4 Recombination detection analysis

Unaligned sequences were analyzed in the SDT v1.2 program, and a pairwise scan was performed with MUSCLE. Data was saved with a minimum identity of 70% and a maximum identity of 100% to ensure sequences were adequately aligned. The aligned CLRDV sequences were then used as an input query and studied for recombination events using Recombination Detection Program (RDP) v 4.0 [37], BOOTSCAN [38], 3SEQ, GENECONV [39], MAXCHI [40], CHIMAERA [41] and SISCAN [42] available in RDP 4 Beta 4.88. Default settings for the different recombination detection methods and a Bonferroni corrected P-value cut-off of 0.05 were used for analysis.

## 3 Results

### 3.1 CLRDV genome structure

The nearly complete genome sequence of six isolates from Georgia and one isolate from Alabama were 5,866nt in length with seven ORFs. The sequences were submitted to GenBank (Table 1). ORF0 that encodes the P0 silencing suppressor protein (28.9kDa) was 785nt in length. ORF1 and ORF1-2 encode replication-associated proteins P1 (70.1kDa), P1-P2 (118.7kDa) was 1,931nt and 3,217nt, respectively, and ORF1-2 is expressed through a ribosomal frameshift at the 5’ end. The intergenic region between ORF 2 and ORF 3a was 70nt. Protein 3a (ORF3a) is expressed through a sub-genomic RNA strategy and has a non-AUG start codon (CTG). The ORF3 which encodes P3 (coat protein; 22.4kDa) is 605nt in length while the ORF4 which express P4 proteins (movement protein; 19.4kDa) was 555nt in length. The ORF4 lies within ORF3 and has a different reading frame. The ORF5, which expresses P5 protein (77.2 kDa) was 2,087nt. The ORF5 overlaps with ORF3 and ORF4 coding regions. The P3, P4, and P5 proteins are expressed through sub-genomic RNA. The 5’ and 3’ end non-coding regions for these isolates were 70 and 151nt, respectively.

### 3.2 Conserved motifs

**Fig 3:**
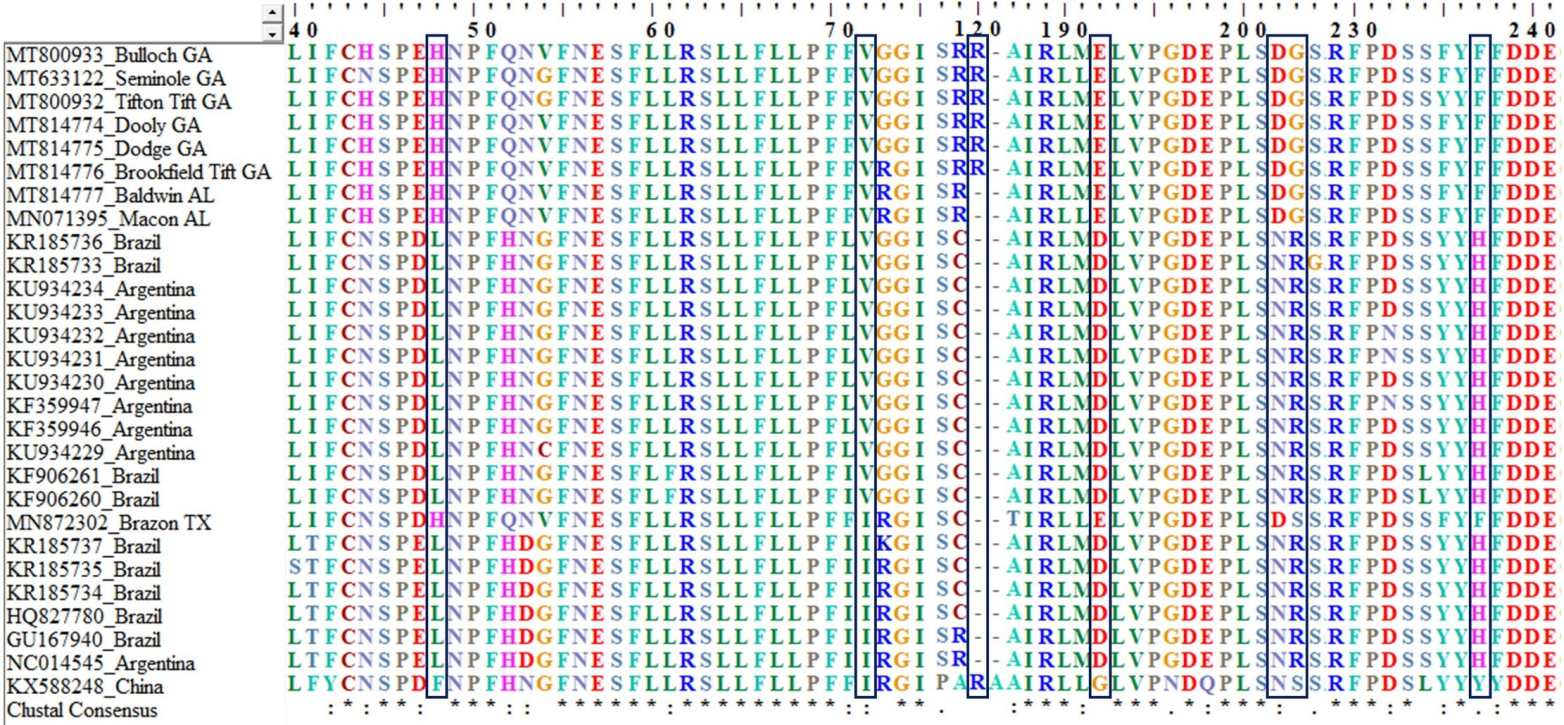
Multiple alignments of a portion of the silencing suppressor protein (P0) showing the conserved motifs and F-box LPxx(L/I) of the cotton leafroll dwarf virus. Only the F-box motif at position 72 and five unique amino acid insertions in Georgia isolates were highlighted with box. The scale represents the position of the unique amino acid within the genome.

The P0 protein of isolated sequences was the most divergent compared to other proteins. In P0, five amino acid substitutions were uniquely present in the CLRDV USA isolates compared to other P0 protein from isolates available in NCBI GenBank. These five unique substitutions are at position 48 [leucine (L)-histidine (H)]; 191 [aspartic acid (D)-glutamic acid (E)]; 201 [asparagine (N)-aspartic acid (D)]; 202 [arginine (R)-glycine (G)]; and 236 [histidine (H)-phenylalanine (F)]. In the F-box LPxx(L/I) motif, all USA isolates sequenced had valine (V) at position 72, except the Texas isolate had isoleucine (I). Therefore, the isolates from Georgia and Alabama resembles the South American resistant-breaking ‘atypical’ genotypes, whereas the Texas isolate resembles the ‘typical’ genotype (Fig 3). At position 120, the insertion of arginine (R) was unique to the isolates from Georgia and China, while was absent in isolates from Alabama, Texas, and South America.

### 3.3 Pairwise identity comparisons

**Fig 4:**
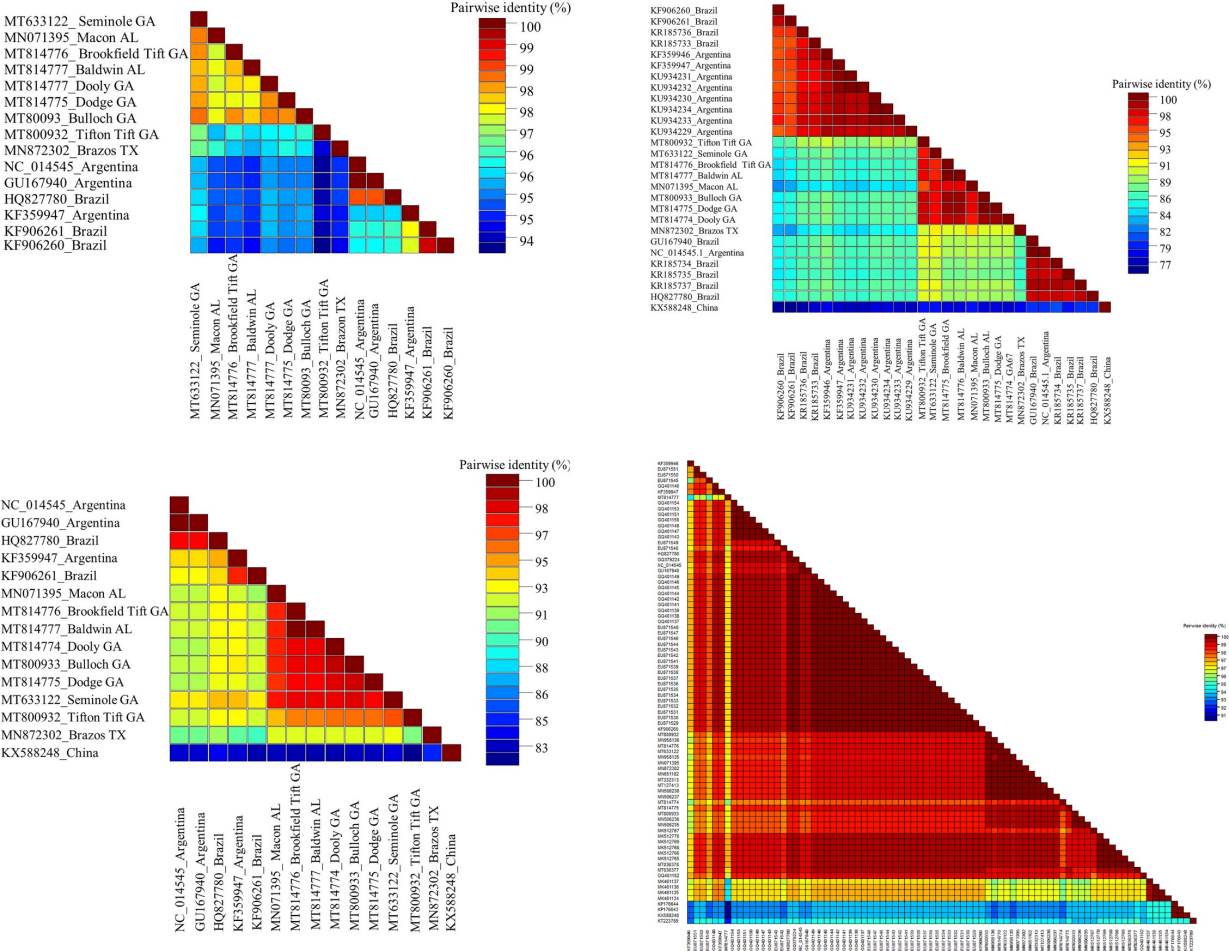
Pairwise identity matrix of Cotton leafroll dwarf virus performed using software SDT v 2.1 (a) Nearly complete nucleotide (b) Silencing suppressor P0 (amino acid) (c) Replication associated protein P1 (amino acid) (d) Partial coat protein P3 (211 nucleotides) sequences from Georgia with sequences available in GenBank.

The sequences of CLRDV isolates from the USA were >94% identical to the South American isolates (Fig 4A). Pairwise identity analysis of the different ORFs of CLRDV isolates sequenced in this study compared to ORFs of GenBank sequences from South America and the USA isolates from Alabama and Texas showed that the ORF0 encoding silencing suppressor protein (P0) was the most divergent (nt 85.75–99.87%; aa 76.54–100%) (Fig 4B). ORF1 encoding P1 protein had 88–100% nucleotide identity (84.27–100% aa identity) (Fig 4C) and was the next most divergent protein. ORF1-2 encoding the fusion protein of P1-P2 had a nucleotide identity of 90.33–100% (80.66–100% aa identity) (S1A Fig). Viral coat protein (P3) encoded by ORF3 had a nucleotide identity of 93.73–100% (94.53–100% aa identity) (S1B Fig). The viral movement protein (P4) encoded by ORF4 had a nucleotide identity of 93.33–100% (86.20–99.43% aa identity) (S1C Fig). ORF 3–5, which encodes aphid transmission and virus accumulation protein (P3-P5) of 77.2kDa, had a nucleotide identity of 90.76–99.09% (91.64–96.54% aa identity) (S1D Fig). Partial ORF3 sequences (211nt) submitted to NCBI GenBank from Arkansas, Florida, Louisiana, Mississippi, South Carolina, and Texas had a nucleotide identity of 90.52–100% (Fig 4D).

### 3.4 Phylogenetic relationships

**Fig 5:**
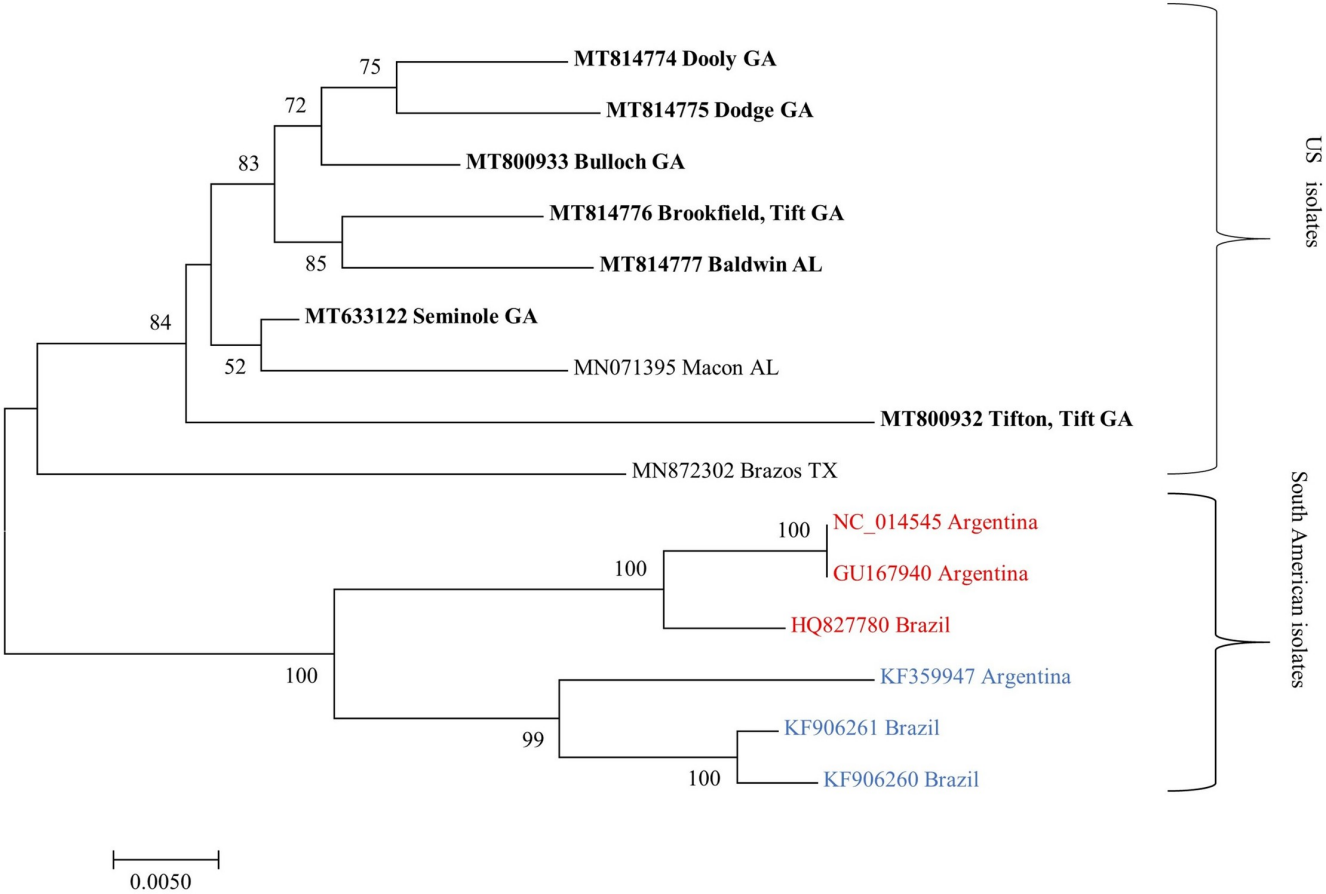
**Maximum likelihood phylogenetic tree of complete nucleotide sequences of cotton leafroll dwarf virus from Georgia (in bold) compared to other isolates from South America represented as typical (red) and atypical (blue) genotypes.** Bootstrap values for 2000 replicates are shown.

The nucleotide sequence of the isolates sequenced from the Alabama, Georgia and Texas formed a separate monophyletic clade that is distinct from South American isolates (Fig 5).

**Fig 6:**
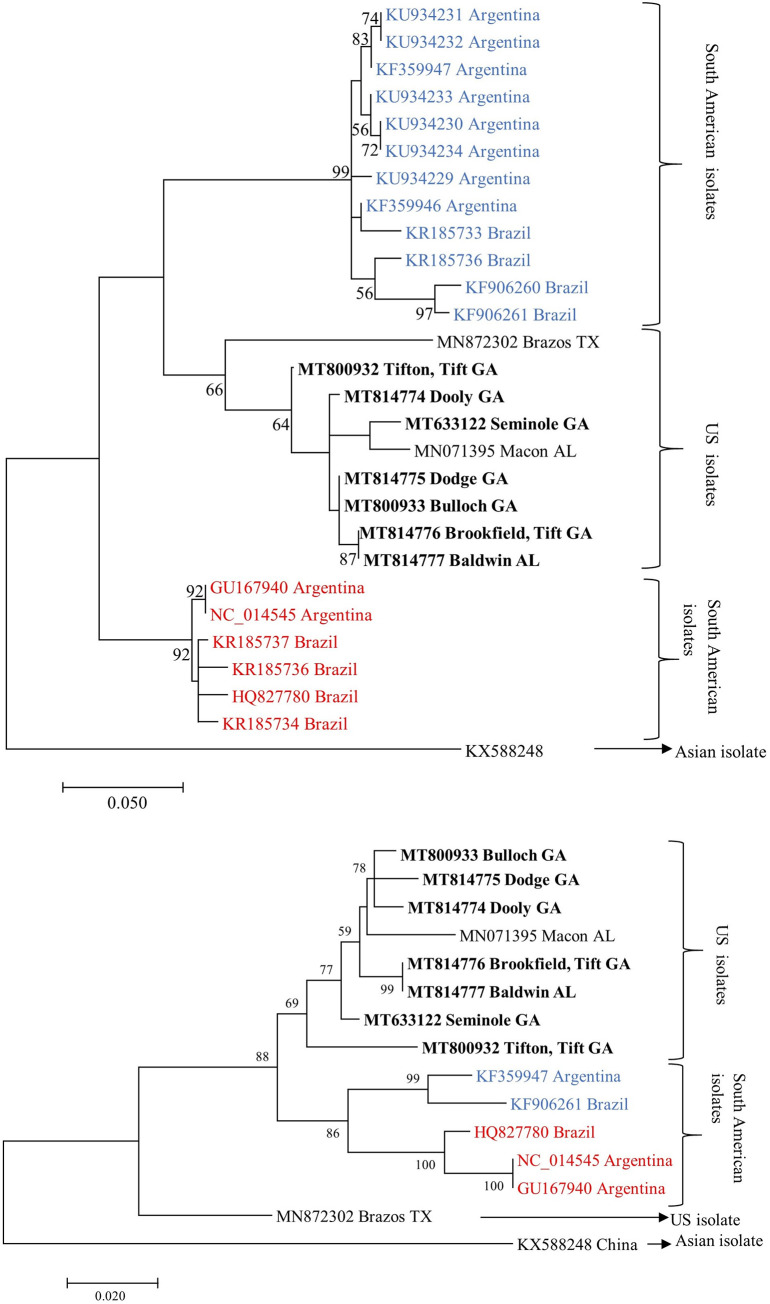
Maximum likelihood phylogenetic tree of amino acid sequences of cotton leafroll dwarf virus (a) Silencing suppressor (P0) and (b) Replication-associated (P1) from Georgia (in bold) compared to other isolates from South America represented as Typical (red) and Atypical (blue) genotypes. Bootstrap values for 2000 replicates are shown.

Among the isolates from the USA, the isolate from Brazos, TX (MN872302) formed a distant clade. The isolate from China (KX588248) sequenced from Soybean aphids (*Aphis glycines*) formed a distant clade in the phylogenetic tree reconstructed based on P0 and P1 amino acid sequences (Fig 6A and 6B). Similar results were produced by phylogenetic trees constructed from nucleotide and amino acid sequences of different ORFs. Only phylogenetic trees derived from amino acid sequences are shown.

**Fig 7:**
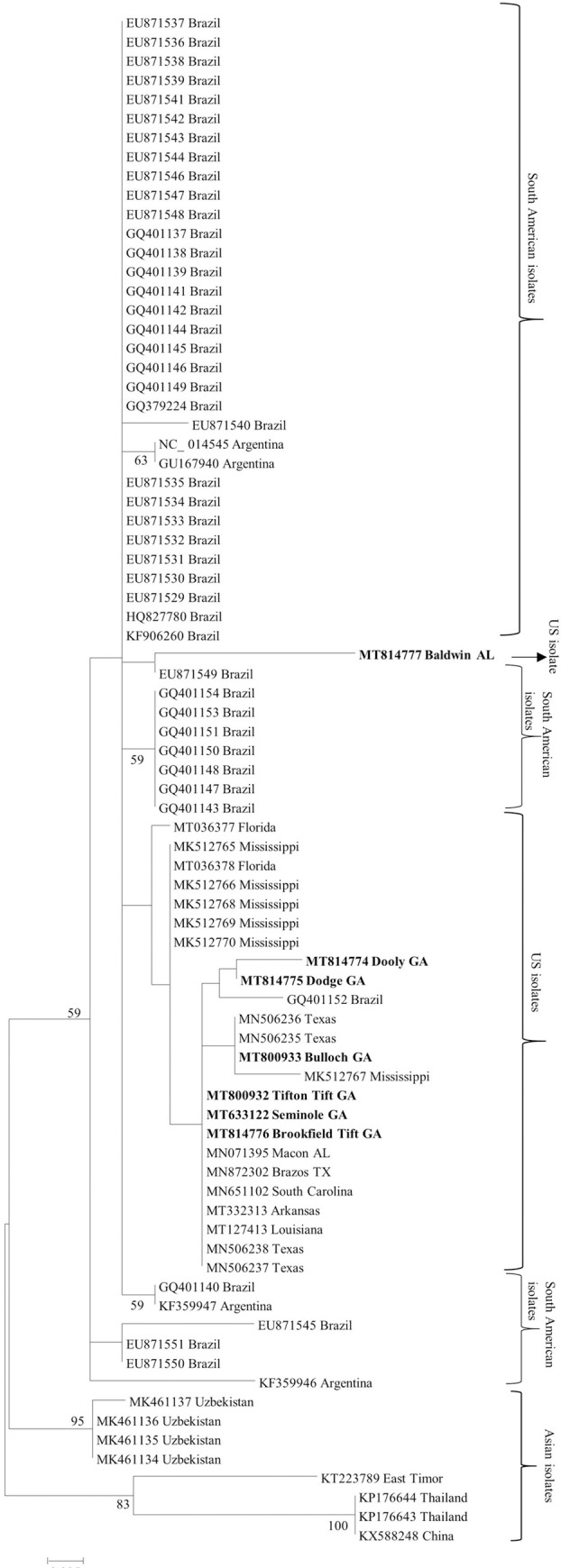
Maximum likelihood phylogenetic tree of partial nucleotide sequences of the coat protein of cotton leafroll dwarf virus from NCBI GenBank compared to Georgia isolates (in bold) from this study. Bootstrap values for 2000 replicates are shown.

Phylogenetic tree based on P3 and P4 amino acid sequences from this study formed a clade closer to South American isolates compared to isolates from Asia (S2A and S2B Fig). Within the USA isolates, Seminole GA (MT633122) and Brazos TX (MN872302) sequences formed a distant clade for P3 protein (S2A Fig). The partial ORF3 sequences (211 nt) from the USA isolates formed a clade with other reported sequences from South America. Within this, the isolate from Baldwin AL (MT814777) was distant from the rest of the US isolates (Fig 7). CLRDV isolates from Asia (KX588248, KP176643, KP176644, MK461134, MK461135, MK461136, MK461137, KT223789) formed distant monophyletic clades (Fig 7). Based on putative amino acid sequences of the P3-P5 protein, Dooly, GA (MT814774), Dodge, GA (MT814775), and Brazos, TX (MN872302) isolates from the USA formed a clade within South American isolates (S2C Fig).

### 3.5 Recombination detection analysis

Ten potential recombination events were detected among the CLRDV full-length genomes (Table 2). Isolates from Tifton, Tift, GA (MT800932), and Dodge, GA (MT814775) had putative parents from Georgia, indicating Georgia isolates are evolving through recombination. Tifton, Tift, GA (MT800932) isolate had recombinant breakpoints in ORF0, whereas Dodge, GA (MT814775) isolate had recombinant breakpoints in ORF1-5. In addition, Tifton, Tift, GA (MT800932) isolate also had a major recombinant parent from Texas and a minor parent from South America, with the recombinant breakpoints were in ORF0. The isolate from Baldwin, AL (MT814777) had two recombination breakpoints at ORF1 and ORF1-3, with parents from Georgia. CLRDV isolate from Macon, AL (MN071395) had a major parent from Brazil (KF906260) and a minor parent from Brookfield, Tift, GA (MT814776), with recombination breakpoints beginning in ORF5 and ending in ORF1. The isolate from Brazos, TX (MN872302) had a major parent from Brazil (HQ8827780) and Seminole, GA (MT633122) as a minor parent with recombination breakpoints beginning in ORF0 and ending in ORF1. At the same time, the Brazilian isolate (KF906260) had a major parent from Argentina (GU167940) and putative minor parents from Georgia with recombinant breakpoints in ORF1. The isolate from Argentina (KF359947) had two recombination events, with one of them having USA isolates as parents. It had recombinant breakpoints beginning in ORF5 and ending in ORF0, and the other one had South American isolates as parents with recombinant breakpoints beginning in ORF1 and ending in ORF5. The recombinant analyses were statistically significant as detected by at least two recombinant programs employed. These results suggest that the CLRDV isolates from the USA have a close genetic relationship and are evolving using a recombination mechanism.

**Table 2:** Predicted recombination events among cotton leafroll dwarf virus isolates sequenced in this study along with those from the GenBank.

Isolate	Parental isolate		Recombination Detection Program	Breakpoint		P-value*
	Major	Minor		Begin	End	
MT800932 (Tifton, Tift, GA)	Unknown (MN872302, Brazos, TX)	KF359947 (Argentina), KF906261 (Brazil), KF906260 (Brazil)	**RDP**, GENECONV, Bootscan, Maxchi, Chimaera, SiSscan, 3Seq	184	772	3.71E-39
MT800932 (Tifton, Tift, GA)	MT633122 (Seminole, GA)	Unknown (MT814776-Brookfield, Tift, GA)	GENECONV, Bootscan, Maxchi, Chimaera, **SiSscan**, 3Seq	1056	3712	2.65E-25
MT814775 (Dodge, GA)	MT80093 (Bulloch, GA)	Unknown (MT814776-Brookfield, Tift, GA)	RDP, Maxchi, Chimaera, **SiSscan**, 3Seq	2645	4306	2.85E-06
MT814777 (Baldwin, AL)	Unknown (MT814776-Brookfield, GA)	MT814775 (Dodge, GA)	Maxchi, **Chimaera**, SiSscan, 3Seq	2912	4662	2.61E-06
MT814777 (Baldwin, AL)	MT814777 (Dooly, GA)	MT800932 (Tifton, Tift, GA)	**GENECONV**, Bootscan, 3seq	1232	1299	9.96E-05
MN071395 (Macon, AL)	KF906260 (Brazil)	MT814776 (Brookfield, Tift, GA)	**GENECONV**, Bootscan, Maxchi, Chimaera, SiSscan, 3Seq	5864	1690	5.06E-75
MN872302 (Brazos, TX)	HQ827780 (Brazil)	MT633122 (Seminole, GA)	**RDP**, Bootscan, Maxchi, Chimaera, 3seq	773	1694	3.08E-31
KF359947 (Argentina)	MN872302 (Brazos, TX)	Unknown (MT814777-Dooly, GA), Unknown (MT800932-Tifton, Tift, GA), Unknown (MT633122-Seminole, GA), Unknown (MT80093-Bulloch, GA), Unknown (MT814775-Dodge, GA), Unknown (MT814776-Brookfield, GA), Unknown (MT814777-Baldwin, AL)	**RDP**, GENECONV, Bootscan, Maxchi, 3seq	5864	183	8.42E-20
KF906260 (Brazil)	GU167940 (Argentina)	Unknown (MT814776-Brookfield, GA), Unknown (MT800932-Tifton, Tift GA), Unknown (MT633122-Seminole, GA), Unknown (MT814777-Dooly, GA), Unknown (MT80093-Bulloch, GA), Unknown (MT814775-Dodge, GA), Unknown (MT814777-Baldwin, AL)	**RDP**, Maxchi, Chimaera, 3seq	918	1622	3.04E-25
KF359947 (Argentina)	KF906261 (Brazil), KF906260 (Brazil)	Unknown (HQ827780-Brazil)	RDP, Maxchi, **Chimaera**, 3seq	2651	5693	8.74E-07

*P-value of the recombinant method is indicated in bold.

## 4 Discussion

Cotton blue disease caused by CLRDV is a major disease of cotton in Africa, Asia, and South America [6] and was recently detected in the cotton-growing belt of the USA. It is essential to understand the symptomatology, epidemiology, mode of transmission, and host-virus-vector interactions for developing disease management strategies. CLRDV and the associated symptoms in the USA are closely related to CBD in South America. In Georgia, the predominant symptoms observed include reddening of leaves, shortened internodes, leaf distortion, downward leaf curling, abnormal top growth, and brittle leaves; however, symptom expression of CLRDV appears to differ among cotton varieties. Some of the symptoms resembled disease caused by soil-borne pathogens and other biotic and abiotic stress, complicating diagnosis-based only on visual symptoms.

In this study, the diversity of CLRDV isolates in Georgia were analyzed and compared with sequences available in GenBank from South America along with two USA isolates from Alabama and Texas. Pairwise sequence identity showed that the P0 protein from Texas and Alabama isolates were >90% identical to Georgia isolates, whereas Georgia isolates were >10% divergent from CLRDV sequences from South America. Poleroviruses reported from Argentina and Brazil showed that the P0 and P1 were the most divergent regions in the genome that contribute to the genetic variability of CLRDV [5, 6, 8]. The isolates present in Asia were more divergent from the USA and South American isolates based on phylogenetic and pairwise identity analyses. According to the ICTV, differences in host-range, serology, one-or two-way cross-protection failure, >10% divergence in the amino acid sequence of any gene product in the Luteoviridae family constitutes a species demarcation in the genus [43].

The silencing suppressor activity of CLRDV is associated with the F-box motif located in the P0 protein. The alignment of CLRDV isolates from Georgia had a substitution of valine (V) from isoleucine (I) at position 72 in the F-box motif (LPxx(L/I), consistent with the substitution found in resistant-breaking CLRDV ‘atypical’ genotypes from South America [8]. The CLRDV isolate from Alabama also had the same substitution as resistant-breaking CLRDV ‘atypical’, whereas Texas isolate had an isoleucine (I) at position 72, similar to CLRDV ‘typical’ genotype. This single amino acid substitution in P0 may not be the only factor contributing to the resistant breaking genotypes in developing different symptoms observed in South America. Additional studies are needed to further confirm the role of this mutation in the ability of break the R gene. The five unique substitutions identified in the P0 protein of CLRDV isolates from the USA could also contribute to different symptoms associated with this disease. Georgia isolates also had a unique insertion of arginine (R) at the 120 position, which was not present in other isolates from the USA or South America. Interestingly, this unique insertion was present in the isolate reported from China (KX588248). The role of this insertion is yet to be determined in disease development. Further studies are needed to understand the role of the unique mutations in the isolates from Georgia for disease development and symptom expression.

The ORFs and proteins encoded by all Georgia isolates had >90% sequence identity with other reported sequences from the USA. Two recombinants were detected in Georgia isolates sequenced in this study with putative parents from Georgia suggesting that the isolate prevalent in Georgia represents a single virus genotype and potentially is evolving through recombination. Georgia isolates were putative parents (major/minor parents) with potential recombinant detected from Alabama, Texas, and other South American isolates. The recombination breakpoints detected were mostly present in ORFs located at 5’ end, predominantly in ORF0 (P0), probably explaining the divergence in the P0 protein. Luteovirus has a conserved 3’ region, and differences are observed in the genome’s 5’ region [3]. For examplr the sugarcane yellow leaf virus in the genus Polerovirus is a recombinant virus with a luteovirus-like capsid and a polerovirus-like polymerase sequence [44]. Our data suggest that CLRDV isolates from the USA are evolving through recombination; however, a broader study involving more isolates is needed to confirm this hypothesis.

Yield losses associated with the typical CDB in susceptible cultivars have been reported up to 80% if cotton aphids were not adequately controlled during the early crop season in South America [6]. In Brazil, the atypical CDB is less aggressive, causing fiber yield loss in susceptible cultivars from 14.6 to 21.5% in fields with a vector threshold level of 80% [45]. Despite the detection of CLRDV in most of the growing areas of the USA, the economic importance and epidemiology of this virus is poorly understood. In a recent survey, CLRDV was detected in both asymptomatic plants from both the commercial fields and research trials. Asymptomatic plants did not show a reduction in yield but could be acting as a virus reservoir. Persistence of CLRDV in cotton basal regrowth and alternate weed hosts such as *Amaranthus* sp., *Lamium amplexicaule*, *Trifolium repens*, *Geranium carolinianum*, *Oenothera* sp., *Stellaria media*, *Gamochaeta purpurea*, *Rumex crispus*, and *Raphanus raphanistrum* have been detected [46, 47]. Thus far, control measures recommended to the growers include weed control and destruction of cotton stalks after harvest [46]. The development of resistant cotton varieties in conjunction with other molecular tools such as gene editing to identify resistant sources against the virus would assist us developing integrated disease management strategies. CLRDV is an emerging virus of cotton in the USA; studies on its impact on fiber quality and yield are the subject of ongoing research.

## Supporting information

S1 Fig Pairwise identity matrix of cotton leafroll dwarf virus from Georgia with other reported amino acid sequences from GenBank. (a) P1-P2 protein; (b) P3 protein; (c) P4 protein; and (d) P3-P5 protein.
(TIF)

S2 Fig Maximum likelihood phylogenetic tree of amino acid sequences of cotton leafroll dwarf virus (a) P3 protein (b) P4 protein and (c) P3-P5 protein from Georgia compared to other sequences from GenBank generated in MEGA X software. Bootstrap values for 2000 replicates are shown.
(TIF)
