# Supplementary figures and images for: Genome analysis of cotton leafroll dwarf virus reveals variability in the silencing suppressor protein, genotypes and genomic recombinants in the USA
Source: PLoS One. 2021 Jul 7;16(7):e0252523. doi: 10.1371/journal.pone.0252523 (PMC8262794; doi:10.1371/journal.pone.0252523)

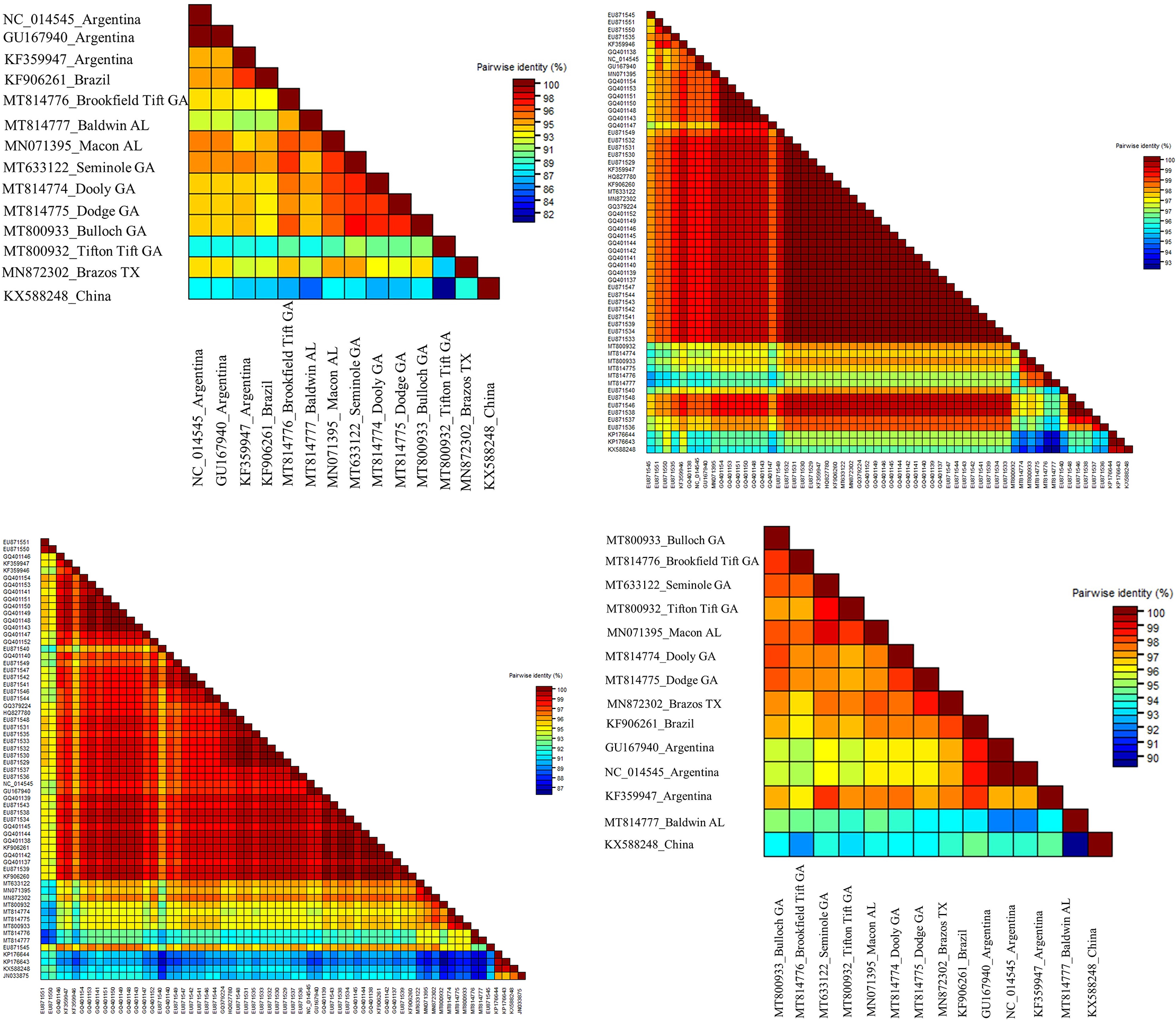

Supplement: S1 Fig — (a) P1-P2 protein; (b) P3 protein; (c) P4 protein; and (d) P3-P5 protein. (TIF) [file pone.0252523.s001.tif]

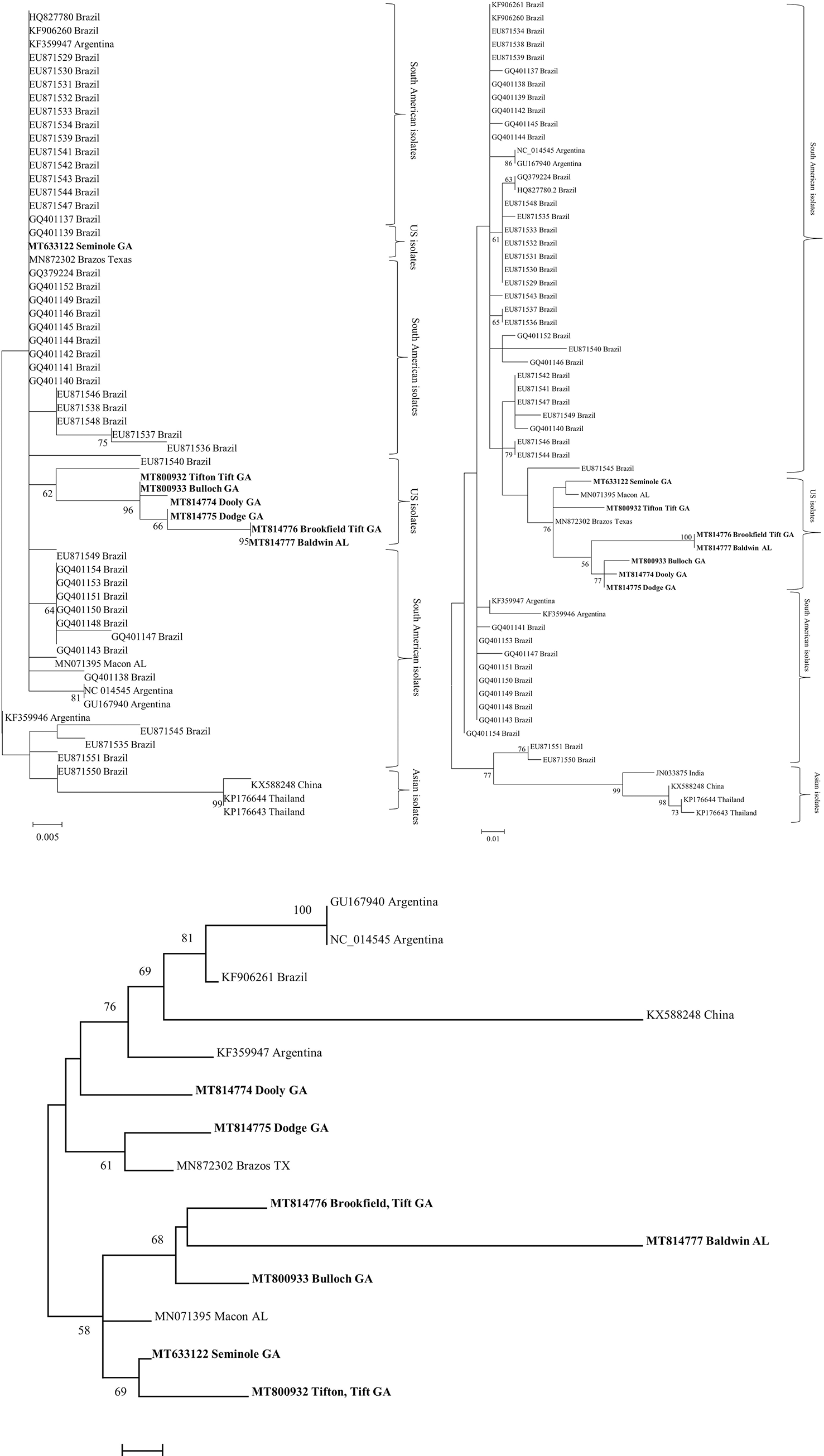

Supplement: S2 Fig — Maximum likelihood phylogenetic tree of amino acid sequences of cotton leafroll dwarf virus (a) P3 protein (b) P4 protein and (c) P3-P5 protein from Georgia compared to other sequences from GenBank generated in MEGA X software. Bootstrap values for 2000 replicates are shown. (TIF) [file pone.0252523.s002.tif]
